# Nitrous oxide as diazo transfer reagent

**DOI:** 10.1039/d4sc04530k

**Published:** 2024-08-14

**Authors:** Alexandre Genoux, Kay Severin

**Affiliations:** a Institut des Sciences et Ingénierie Chimiques, École Polytechnique Fédérale de Lausanne (EPFL) 1015 Lausanne Switzerland kay.severin@epfl.ch

## Abstract

Nitrous oxide, commonly known as “laughing gas”, is formed as a by-product in several industrial processes. It is also readily available by thermal decomposition of ammonium nitrate. Traditionally, the chemical valorization of N_2_O is achieved *via* oxidation chemistry, where N_2_O acts as a selective oxygen atom transfer reagent. Recent results have shown that N_2_O can also function as an efficient diazo transfer reagent. Synthetically useful methods for synthesizing triazenes, N-heterocycles, and azo- or diazo compounds were developed. This review article summarizes significant advancements in this emerging field.

## Abstract 2

The review summarizes the use of nitrous oxide (N_2_O, ‘laughing gas’) as a diazo transfer reagent in synthetic chemistry.

## Graphical abstract

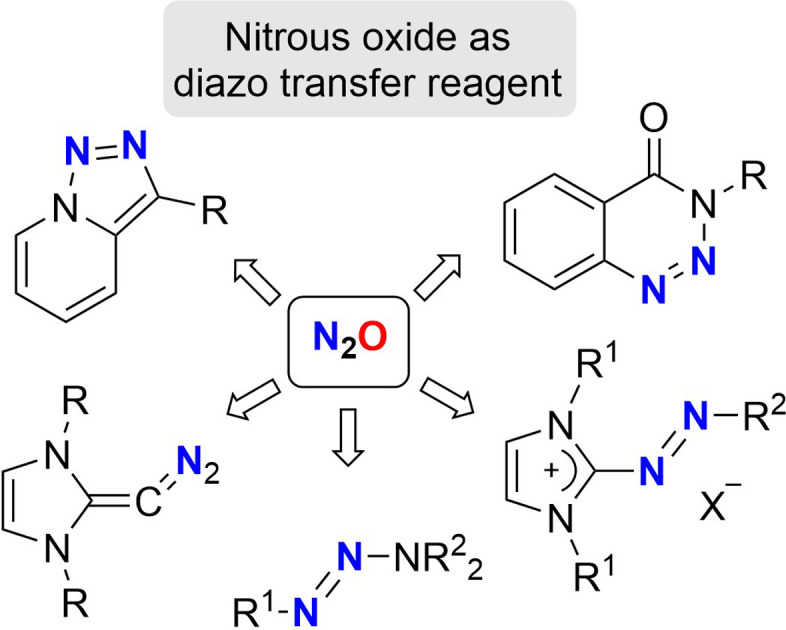

## 1. Introduction

Nitrous oxide was brought to the public's attention by Sir Humphry Davy, an influential British chemist and inventor. In 1800, the 21-year-old Davy published a book entitled “*Researches, Chemical and Philosophical; Chiefly Concerning Nitrous Oxide, or Dephlogisticated Nitrous Air, and its Respiration*”.^1^ This 580-page monograph is divided into two parts. The first part provides a comprehensive review of the chemistry of nitrous oxide, summarizing the state of knowledge at the time. The second part explores the physiological effects of nitrous oxide with Davy giving detailed descriptions of the sensations caused by inhaling this gas. The book also summarizes the effects of nitrous oxide on various animals. Davy was fascinated by this gas, and his enthusiasm was contagious. As a result, nitrous oxide quickly became a popular recreational drug among the British upper class.

**Scheme 1:**
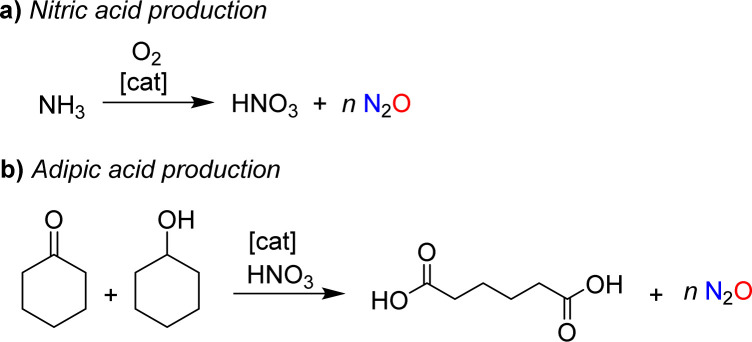
Nitrous oxide is formed as a side product during the industrial production of nitric acid (a) and adipic acid (b).

The use of nitrous oxide as a drug continues to make headlines today,^2^ but other concerns have emerged. Nitrous oxide is a potent greenhouse gas (GWP = 300), contributing significantly to global warming.^3^ Furthermore, it is an ozone-depleting substance.^4^ Various anthropogenic sources contribute to N_2_O emissions, many of which are linked to agriculture.^5^ However, mitigation strategies have primarily focused on industrial processes, where nitrous oxide is formed as a side product.^6^ The largest amount of industrial N_2_O is generated during the production of nitric acid (Scheme 1a).^7^ N_2_O is produced alongside the desired NO during the catalytic oxidation of ammonia, with the amount of N_2_O depending on the process conditions. Plants without abatement technologies are estimated to emit between 4 and 19 kg of N_2_O per ton of HNO_3_ (100%).^7*b*^ Another significant source of nitrous oxide is the production of adipic acid.^8^ Adipic acid is obtained through the catalytic oxidation of a mixture of cyclohexanone and cyclohexanol with nitric acid, resulting in the formation of approximately 300 kg of N_2_O per ton of adipic acid (Scheme 1b).

While the formation of N_2_O during the production of nitric and adipic acid is a major concern, it also presents an opportunity. Some companies have developed processes that allow the isolation of N_2_O.^9^ Nitrous oxide can then be sold, or used as a reagent in downstream applications.^10^

**Scheme 2:**
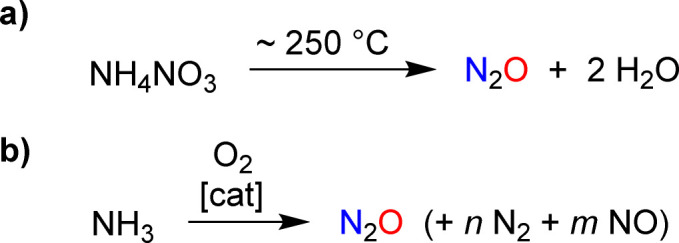
Synthesis of nitrous oxide by thermal decomposition of ammonium nitrate (a) or by catalytic oxidation of ammonia (b).

The targeted synthesis of nitrous oxide is achieved through the thermal decomposition of a concentrated ammonium nitrate solution (Scheme 2a).^11^ This process is performed on an industrial scale. However, it is not ideal because the production of NH_4_NO_3_ involves a multi-step manufacturing route. An interesting alternative is the direct oxidation of ammonia with a catalytic system that provides high selectivity for N_2_O over NO and N_2_ (Scheme 2b).^12^ Pilot tests^12^ and advances in catalyst design^13^ suggest that ammonia oxidation could become a feasible method for large-scale industrial N_2_O production.

The chemical valorization of nitrous oxide is traditionally achieved *via* oxidation reactions.^12,14–16^ N_2_O is a powerful oxidant from a thermodynamic standpoint,^14^ and the by-product, N_2_, is both easy to separate and harmless. Furthermore, N_2_O displays good solubility in organic solvents, enabling liquid-phase reactions in low-polarity media.^15^ A drawback of N_2_O as an oxidant is its kinetically inert nature. However, the high kinetic barrier can be overcome by using a catalyst and/or elevated temperatures and pressures.

**Scheme 3:**
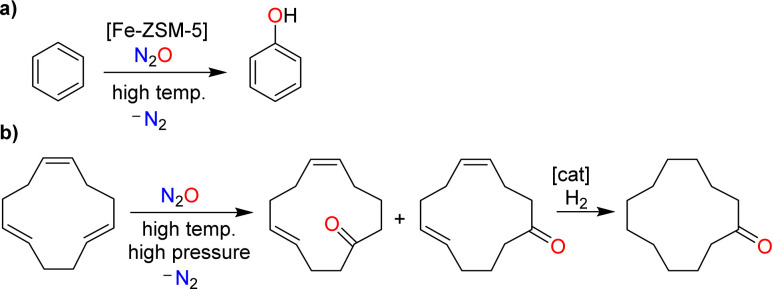
The use of nitrous oxide as an O-atom donor: synthesis of phenol by catalytic oxidation of benzene (a), and synthesis of cyclododecanone by non-catalytic oxidation of 1,5,9-cyclododecatriene, followed by hydrogenation (b).

An example of a catalytic process involving N_2_O is the hydroxylation of benzene (Scheme 3a).^8*b*,12^ This reaction is catalyzed by iron-containing zeolites. Nitrous oxide represents an interesting oxidant for this reaction because it provides phenol with high selectivity. Furthermore, one could couple the N_2_O-mediated phenol production with the N_2_O-liberating formation of adipic acid (phenol could be hydrogenated to give cyclohexanol, the precursor for adipic acid; see Scheme 1). The pilot-scale production of phenol using N_2_O as the oxidant has been realized by Solutia, together with the Boreskov Institute of Catalysis.^8*b*,12^ The process has not yet been commercialized due to economic reasons, and because a circular phenol/adipic acid production would require additional N_2_O.^8*b*,12^

The non-catalyzed oxidation of olefins using N_2_O at elevated temperatures and pressures gives ketones alongside N_2_.^15^ This type of reactivity forms the basis for the industrial production of cyclododecanone, as developed by BASF.^10^ The process starts with the oxidation of 1,5,9-cyclododecatriene with N_2_O to give cyclododeca-4,8-dien-1-one as a mixture of isomers (Scheme 3b). Catalytic hydrogenation then provides the target cyclododecanone. It is worth noting that the N_2_O, which is used in this process, is obtained from the production of adipic acid.^10^

The reactions depicted in Scheme 3 demonstrate that nitrous oxide can be employed for the synthesis of bulk chemicals. However, many reports about the use of N_2_O as an oxidant pertain to small-scale syntheses, typically conducted in academic settings.^17–22^

For the oxidation of highly reactive compounds, the inert nature of N_2_O can be advantageous, as it prevents potential overoxidation reactions. For example, N_2_O is frequently used for the oxidation of reactive main-group element compounds.^18^ These reactions are typically performed in solution using atmospheric pressure of N_2_O.

The chemical activation of N_2_O under mild conditions can also be achieved with certain transition metal complexes.^19,20^ This capability has spurred efforts to develop reactions with N_2_O using homogeneous catalysts. Over the past few years, significant progress has been made in this field, with efficient catalysts being developed for a variety of oxidation reactions.^21,22^

**Scheme 4:**
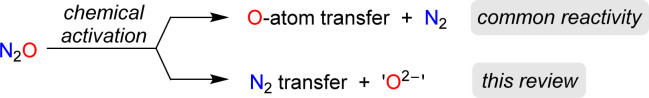
Upon chemical activation, nitrous oxide typically acts as O-atom donor. This review focuses on reactions in which N_2_O functions as diazo transfer reagent.

Most of the reactions discussed thus far proceed *via* oxygen atom transfer and extrusion of dinitrogen. This review focuses on a different type of reactivity, namely the use of N_2_O as a diazo transfer reagent (Scheme 4). The formal by-product in these reactions is O^2−^, which is released in the form of hydroxide, alkoxide, oxide salts (M^I^OH, M^I^OR, M^II^O), or water, depending on the substrate that was employed.

The use of N_2_O as a diazo transfer reagent was first demonstrated by Wislicenus in 1892.^23^ By subjecting NaNH_2_ to N_2_O at elevated temperatures, he was able to obtain NaN_3_. The ‘Wislicenus reaction’ is nowadays employed for the industrial production of NaN_3_.^24^ Despite this early success, N_2_O-based diazo transfer reactions have historically remained underdeveloped. However, significant progress has been made in recent years, resulting in numerous synthetically useful processes. This review summarized significant developments in this area. Before discussing these advancements, we will describe the covalent capture of intact N_2_O by (semi-)metal complexes, frustrated Lewis pairs (FLPs), and organic nucleophiles. Only a few of these adducts were used in productive diazo transfer reactions, but they provide valuable insights into the underlying reactivity of N_2_O.

## 2. Covalent capture of nitrous oxide

**Scheme 5:**
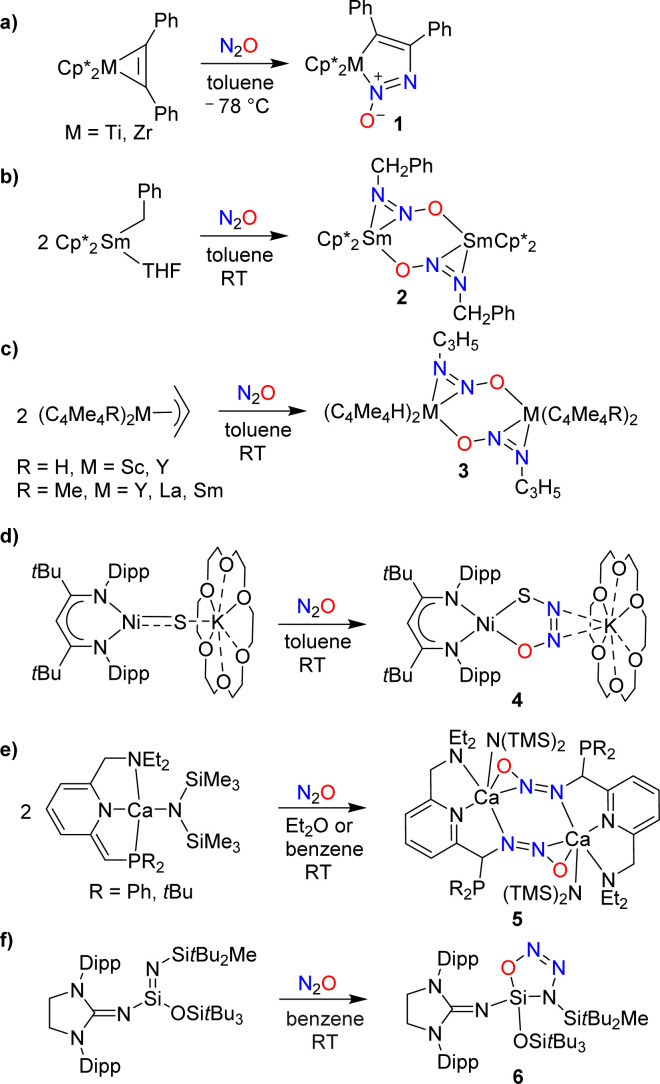
Cooperative covalent capture of N_2_O by (semi-)metal complexes (Dipp = 2,6-C_6_H_3_iPr_2_).

The covalent trapping of N_2_O by (semi-)metal complexes leverages metal–ligand cooperation.^25–33^ A common theme in these reactions is the formation of a covalent bond at the terminal N-atom of N_2_O along with a coordination bond to the other O/N-atom (Scheme 5a–f). Nitrous oxide can also act as a simple ligand for metal complexes (without concomitant formation of a covalent bond to a main group element), but these cases will not be discussed further in this review.^34^

Hillhouse and coworkers have investigated reactions of the diphenylacetylene complexes Cp*M(PhC_2_Ph) (M = Ti, Zr) with N_2_O.^25^ At low temperatures, azoxymetallacyclopentene complexes of type 1 were obtained (Scheme 5a). The zirconium complex was found to be thermally labile, undergoing extrusion of N_2_ upon warming to room temperature. The titanium complex was more stable, allowing for a crystallographic characterization. More recently, it was found that the zirconium complex can be stabilized by *N*-alkylation with MeOTf.^26^

Insertion of N_2_O into a metal–carbon bond was also observed for samarium complexes. When a solution of (Cp*)_2_SmBn(THF) in toluene was exposed to N_2_O, the dinuclear complex 2 was formed (Scheme 5b).^27^ Allyl complexes of the general formula (C_5_Me_5_R)_2_M(C_3_H_5_) (R = H, Me; M = Sc, Y, Sm, La) were found to display a similar reactivity (Scheme 5c).^28^

Hayton and coworkers reported that a “masked” terminal Ni(ii) sulfide complex is able to react with N_2_O to give the thiohyponitrite complex 4 (Scheme 5d).^29^ Liberation of N_2_ was observed when a solution of complex 4 was heated in toluene at 45 °C for 6 days, resulting in the formation of a η^2^-SO complex as the main product.^30^ More recently, the Hayton group showed that a Zn(ii) sulfide analogous to complex 4 is also converted to a thiohyponitrite complex when exposed to N_2_O.^31^

The cooperative metal–ligand activation of N_2_O is not restricted to transition metal complexes. Milstein and coworkers examined the reaction of N_2_O with dearomatized calcium pincer complexes supported by pyridine-based PNN-type ligands.^32^ A rapid transformation into dinuclear diazotate complexes (5) was observed at room temperature (Scheme 5e).

The reactions of low-valent silicon compounds with N_2_O typically proceed *via* O-atom transfer and liberation of dinitrogen.^18^ An exception to this reactivity pattern was reported by Inoue and coworkers. They showed that an oxatriazasilole, 6, is formed upon reaction of a silaimine with N_2_O (Scheme 5f).^33^ The reaction proceeds *via* a concerted 1,3-dipolar cycloaddition mechanism, first proposed by Wiberg,^35^ and later supported by computational studies.^36^ It is interesting to note that solutions of the cycloaddition product 6 are thermally very stable; no isomerization or decomposition was observed at temperatures up to 130 °C.^33^

**Scheme 6:**
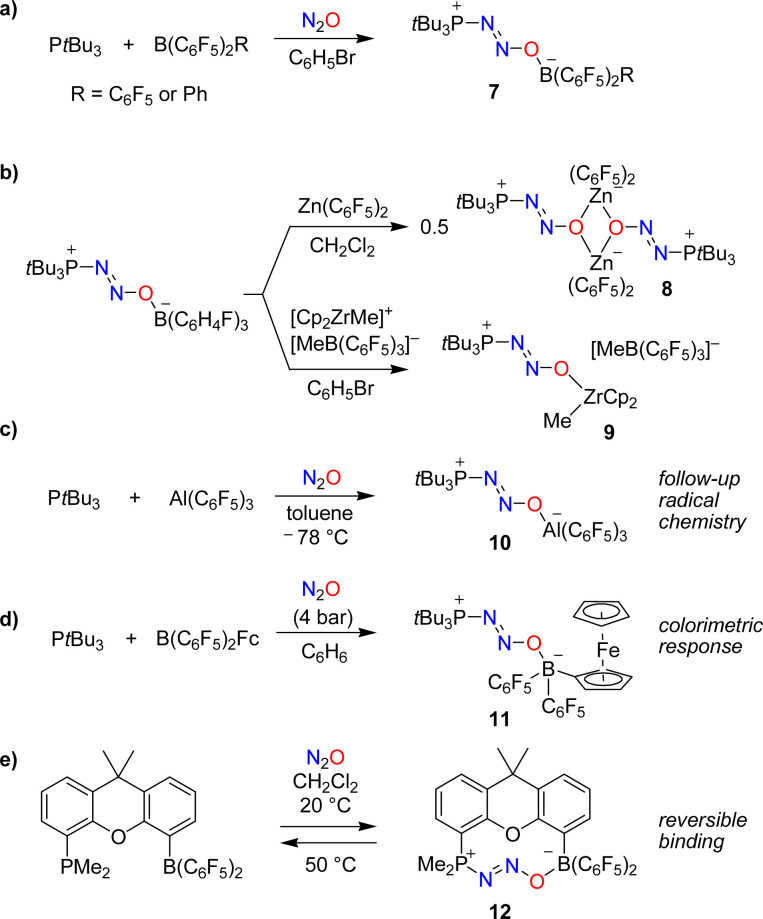
Covalent capture of nitrous oxide by frustrated Lewis pairs (FLPs).

The utilization of FLPs for the capture of N_2_O was first investigated by Stephan and coworkers.^37,38^ When mixtures of the bulky phosphine P*t*Bu_3_ and the Lewis acids B(C_6_F_5_)_2_R (R = C_6_F_5_ or Ph) were exposed to an atmosphere of N_2_O, zwitterionic adducts of type 7 with P–N_2_O–B linkages were obtained (Scheme 6a). The thermal or photochemical activation of 7 resulted in the formation of (*t*Bu_3_PO)B(C_6_F_5_)_2_R along with the liberation of dinitrogen.

In follow-up studies, the Stephan group showed that the boron-based Lewis acid can be varied widely to give adducts of the general formula ‘*t*Bu_3_P(N_2_O)BR_2_R’.^39–41^ In contrast, phosphines with reduced steric hindrance or Lewis basicity do not form similar compounds. The borane can be exchanged for other Lewis acids. The adduct *t*Bu_3_P(N_2_O)B(C_6_H_4_F)_3_ is particularly well suited for exchange reactions because it contains the relatively weak Lewis acid B(C_6_H_4_F)_3_. For example, the dinuclear complex 8 was obtained when adding one equivalent of Zn(C_6_F_5_)_2_,^39^ whereas exchange with [Cp_2_ZrMe][MeB(C_6_F_5_)_3_] gave complex 9 (Scheme 6b).^40^

The capture of N_2_O can also be achieved by using an alane. The adduct *t*Bu_3_P(N_2_O)Al(C_6_F_5_)_3_ (10) was obtained by slow addition of N_2_O to a cooled solution containing P*t*Bu_3_ (2 eq.) and Al(C_6_F_5_)_3_(toluene) (Scheme 6c).^42^ The reaction with additional Al(C_6_F_5_)_3_(toluene) resulted in N–O bond rupture, generating the highly reactive radical ion pair (*t*Bu_3_P˙)[(C_6_F_5_)_3_Al(O˙)Al(C_6_F_5_)_3_] that can activate C–H bonds.

The colorimetric detection of N_2_O was realized using a borane with a ferrocenyl (Fc) substituent as the Lewis acid in an FLP.^43^ Exposing a mixture of P*t*Bu_3_ and B(C_6_F_5_)_2_Fc to N_2_O resulted in the formation of the adduct *t*Bu_3_P(N_2_O)B(C_6_F_5_)_2_Fc (11), accompanied by a color change from maroon to amber (Scheme 6d). A different UV-Vis-responsive FLP was created by using a phosphine containing a cycloheptatrienyl-cyclopentadienyl titanium sandwich complex as substituent.^44^

The use of a single-component FLP with a dimethylxanthene backbone allowed for the reversible binding of N_2_O.^45^ Exposing a solution of this FLP in dichloromethane to one atmosphere of N_2_O resulted in the slow (*t*_1/2_ ∼ 12 h) formation of the adduct 12 (Scheme 6e). Warming a solution of this adduct in dichloromethane to 50 °C for 2 h led to the quantitative removal of N_2_O.

**Scheme 7:**
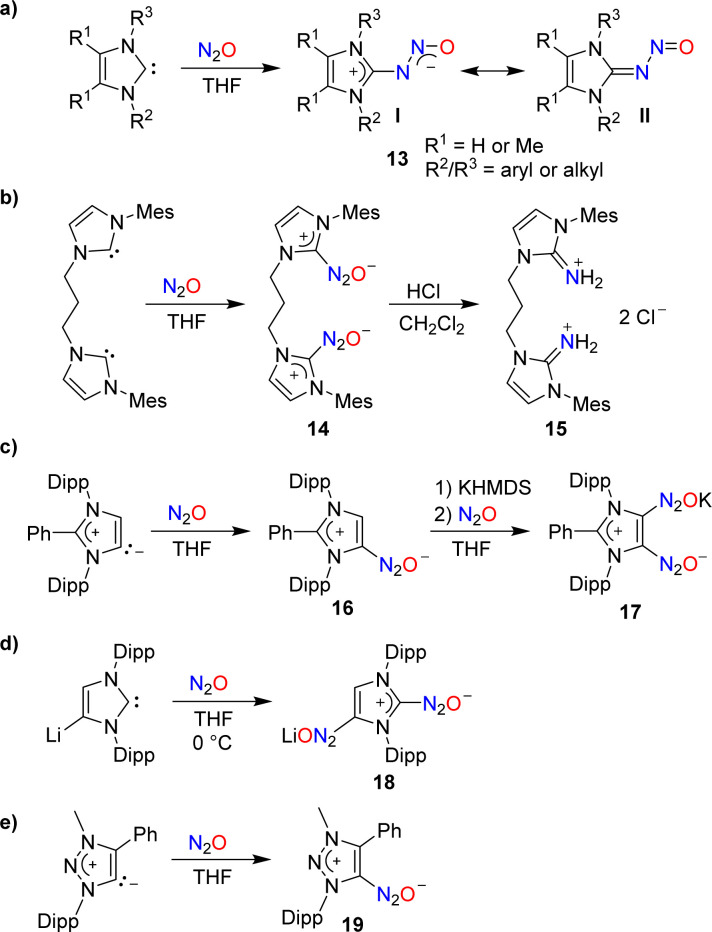
Covalent capture of N_2_O by N-heterocyclic carbenes (NHCs).

In 2012, our group demonstrated that N-heterocyclic carbenes (NHCs) can effectively capture N_2_O.^46^ When a solution of 1,3-dimesitylimidazol-2-ylidene (IMes) in THF was subjected to one atmosphere of N_2_O, the adduct IMes(N_2_O) was formed in high yield (90%). A similar compound was obtained using an imidazole-2-ylidene with Dipp wingtip groups (IPr). Subsequent studies by our group and by others showed that N_2_O adducts of the general formula NHC(N_2_O) (13) are accessible with a range of different substituents on the heterocycle (Scheme 7a).^47–49^ The adducts can be described as zwitterionic imidazolium diazotates (13, I) or as nitrosoimines (13, II). Crystallographic analyses revealed a preference for a *trans* configuration for the N–N bond, even though exceptions have been reported.^48^ NHC(N_2_O) adducts display good stability at room temperature. At elevated temperatures, the release of N_2_ and formation of the corresponding ureas was observed.

The addition of Brønsted acids to IMes(N_2_O) resulted in the rupture of the N–N bond and the formation of N-heterocyclic iminium salts.^47^ This type of reactivity was used by Dielmann and coworkers for the synthesis of the dimer 15 (Scheme 7b).^50^ The latter was employed as a precursor for the synthesis of a chelate ligand.

N–N bond cleavage was also observed when IMes(N_2_O) was combined with nickel(0)^51^ or cobalt(i)^52^ complexes. A different type of reactivity was noted in reactions with vanadium(iii)^53,54^ and uranium(iii)^55^ complexes. Here, NHC(N_2_O) adducts served as mild O-atom donors.

Similar to Arduengo-type NHCs, mesoionic carbenes derived from C2-arylated 1,3-bis(2,6-diisopropylphenyl)imidazole-2-ylidene are able to form adducts with N_2_O (Scheme 7c).^56^ Interestingly, it was possible to introduce a second N_2_O group by treating adduct 16 with first potassium hexamethyldisilazide (KHMDS) and then N_2_O.

A direct double functionalization with two N_2_O groups was observed when a solution of lithiated IPr in THF was subjected to an atmosphere of N_2_O (Scheme 7d).^56^

N_2_O capture can also be achieved by triazole-based carbenes: the triazolium diazotate 19 was isolated in 86% yield from a reaction of the corresponding carbene with N_2_O (Scheme 7e).^57^

**Scheme 8:**
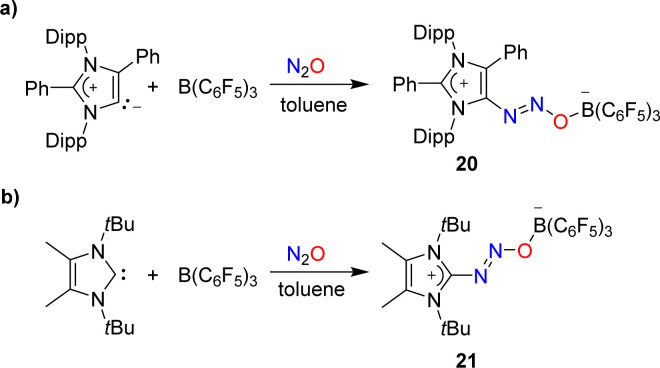
Covalent capture of N_2_O by mixtures of N-heterocyclic carbenes and B(C_6_F_5_)_3_.

Attempts to capture N_2_O with a mesoionic carbene featuring phenyl substituents at the 2- and the 4-position were unsuccessful. However, in the presence of B(C_6_F_5_)_3_, the C–N_2_O–B-bridged adduct 20 was isolated (Scheme 8a).^58^ A similar situation was encountered with a carbene having *tert*-butyl wingtip groups and methyl substituents in 4/5-position: while direct N_2_O capture by the carbene could not be achieved, an adduct (21) was obtained in the presence of the Lewis acid B(C_6_F_5_)_3_ (Scheme 8b).^49^

**Scheme 9:**
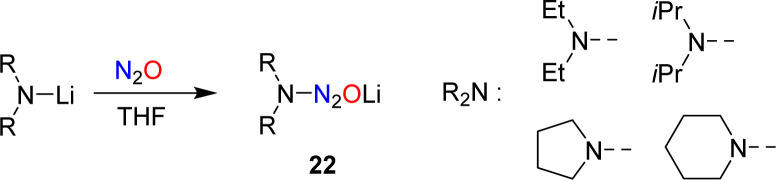
Covalent capture of N_2_O by lithium amides.

In 1953, Meier reported that lithiated amines react with N_2_O.^59^ In the case of Et_2_NLi, he was able to isolate tetraethyltetrazene, albeit in low yield. Meier proposed aminodiazotates as intermediates, but the isolation of these adducts was not attempted. Our group has re-investigated this type of reaction and found that aminodiazotates (22) are formed in good yields when solutions of lithium amides in THF are subjected to an atmosphere of N_2_O (Scheme 9).^60^ N_2_O adducts of type 22 can serve as precursors for the synthesis of triazenes, and more details about such transformations are given in Section 4.

## 3. Synthesis of azides

The standard procedure for the synthesis of NaN_3_ involves the reaction between NaNH_2_ and N_2_O (see Section 1).^23,24^ Meier showed that this chemistry can be extended to organic azides. He noted that a pale yellow oil, most likely phenyl azide, was formed in low yield when a solution of lithiated aniline in diethyl ether was exposed to N_2_O.^59^

**Scheme 10:**
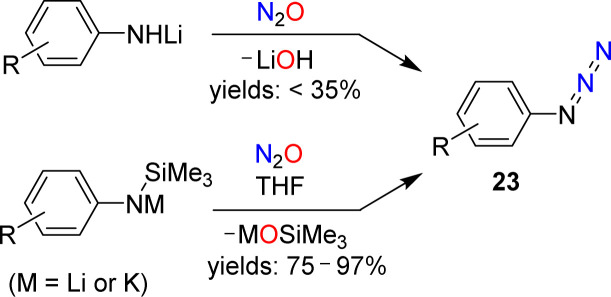
Synthesis of aryl azides.

A more detailed investigation was conducted by Koga and Anselme in 1968.^61^ They showed that aryl azides (23) are formed by reactions of lithiated aromatic amines with N_2_O (Scheme 10). However, the yields of these diazo transfer reactions were poor (<35%). Significantly higher yields were obtained when silylated aryl amides were used as starting materials in NMR-scale reactions.^62^ When the reactions were performed on a preparative scale, an increased amount of side products was observed.

## 4. Synthesis of triazenes

Aromatic triazenes of the general formula (aryl)N_3_R_2_ have been investigated extensively in the context of synthetic organic chemistry.^63^ An important feature of aryl triazenes is the possibility to replace the N_3_R_2_ group under acidic conditions by a broad range of other functionalities. The substitution reactions proceed *via* diazonium compounds, and aryl triazenes are often referred to as “masked diazonium salts”.^64^

**Scheme 11:**
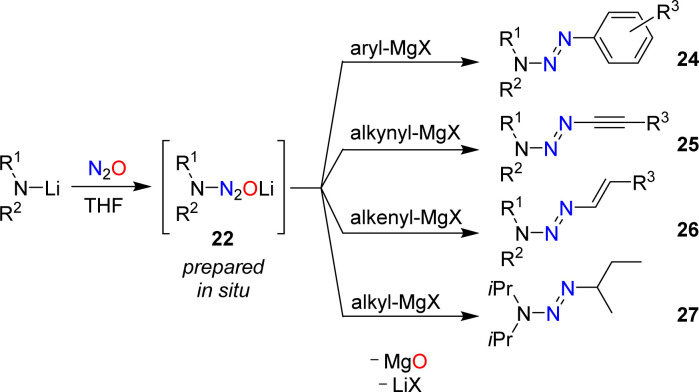
Synthesis of triazenes by reactions of N_2_O-derived aminodiazotates with Grignard reagents.

Aryl triazenes of type (aryl)N_3_R_2_ are typically prepared by coupling of aryldiazonium salts with secondary amines.^63^ In 2015, our group reported an alternative synthetic procedure involving nitrous oxide.^60^ Solutions of lithium amides in THF were allowed to react with N_2_O, resulting in the formation of aminodiazotates (22). The latter were not isolated,^65^ but combined directly with aryl Grignard reagents to give aryl triazenes of type 24 (Scheme 11).

A key advantage of the N_2_O-based methodology for synthesizing triazenes is that it can be extended to alkynyl (25) and alkenyl triazenes (26). These compounds are difficult to access by alternative procedures.^66,67^ Alkyl triazenes can also be prepared by this method, as illustrated by the synthesis of 1-isobutyl-3,3-diisopropyltriazene (27). However, the yield of 27 was low (12%).

**Scheme 12:**
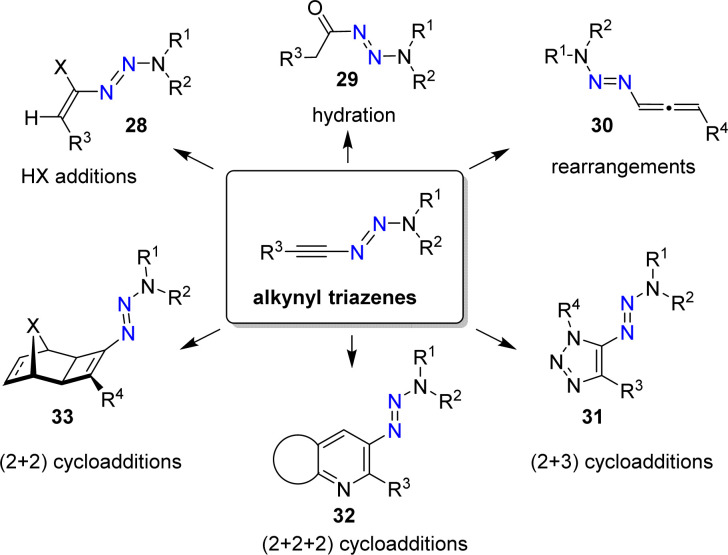
Alkynyl triazenes as versatile starting materials in synthetic organic chemistry.

Alkynyl triazenes are attractive starting materials for application in organic synthesis (Scheme 12).^66^ From a practical standpoint, it is worth noting that alkynyl triazenes are not particularly sensitive to air or moisture. Furthermore, they can be purified by chromatography, and they exhibit good thermal stability.

The N_3_R_2_ group is electron-donating, resulting in an ynamide-like reactivity for alkynyl triazenes. For example, it is possible to perform addition reactions with Brønsted acids to give alkenyl triazenes of type 28.^68,69^ The acid-catalyzed hydration of alkynyl triazenes provides acyl triazenes (29),^70^ and allenyl triazenes of type 30 are accessible by base-induced rearrangements.^71^

Alkynyl triazenes are suitable substrates for transition metal-catalyzed cycloaddition reactions. Cui and coworkers have prepared a wide range of triazoles (31) by Ir-catalyzed (2 + 3) cycloaddition reactions of alkynyl triazenes and organic azides (Scheme 12).^72^ In our group, we have used Ru-catalyzed cycloaddition reactions for synthesizing densely functionalized arenes and pyridines (32),^73^ as well as cyclobutenyl triazenes (33).^74^ Further transformations of alkynyl triazenes include Rh-catalyzed annulation reactions,^75–77^ Au-catalyzed cyclizations,^78,79^ Pd-catalyzed addition reactions,^80^ Ficini-type reactions,^81^ light-induced isomerizations,^82^ and electrophilic fluorinations.^83^

An interesting aspect of using alkynyl triazenes as substrates in these reactions is the possibility of performing post-synthetic substitution reactions. For example, the triazene group in pyridines of type 32 can be substituted by a wide range of nucleophiles, including fluoride.

**Scheme 13:**
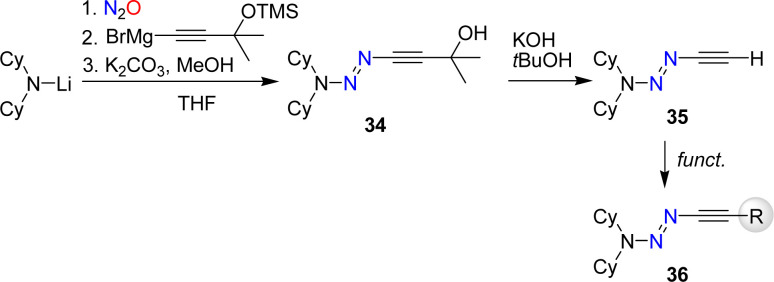
Synthesis of a terminal alkynyl triazene and its functionalization.

The synthesis of alkynyl triazenes by coupling of lithium amides first with nitrous oxide and then with an alkynyl Grignard reagent restricts the functional groups that can be employed. To overcome this limitation, we have developed a synthetic route for a terminal alkynyl triazene, 35 (Scheme 13).^84^ Subsequent functionalization of 35 allowed to prepare alkynyl triazenes with a range of functional groups including esters, alcohols, cyanides, phosphonates, and amides.

## 5. Synthesis of N-heterocycles

**Scheme 14:**
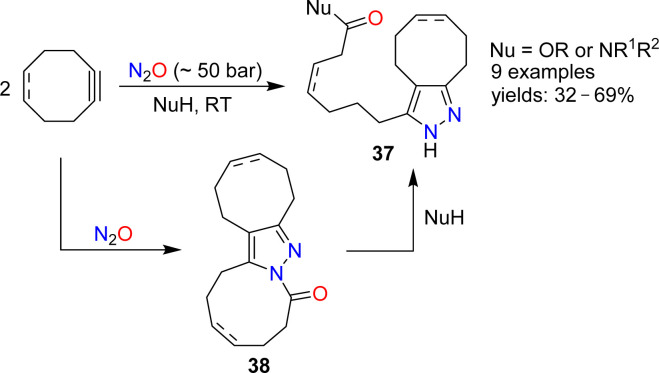
Synthesis of pyrazoles.

The reactions of alkenes and alkynes with N_2_O generally proceed *via* O-atom transfer and liberation of dinitrogen.^15^ An interesting exception to this type of reactivity was reported by Banert and Plefka.^85^ When cyclooctyne or cycloocten-5-yne were treated with nitrous oxide (∼50 bar) in the presence of nucleophiles (amines or alcohols), the formation of pyrazoles of type 37 was observed (Scheme 14). The reactions were proposed to proceed *via* heterocycles of type 38. In the case of cyclooctyne, the intermediate could be isolated if the reaction was performed without nucleophiles.

**Scheme 15:**
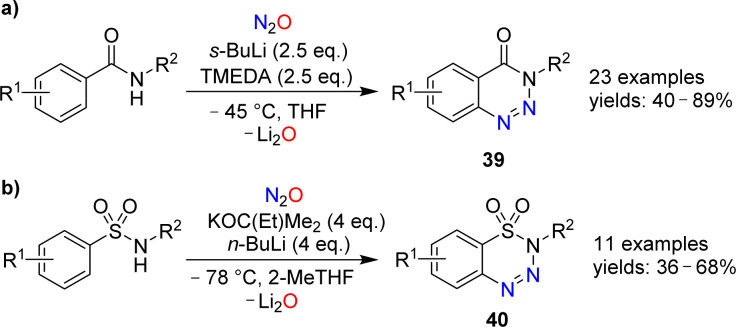
Syntheses of benzotriazines.

Cui and coworkers have shown that nitrous oxide can be used for the synthesis of benzotriazines.^86^ Aromatic amides or sulfonamides were deprotonated with strong bases and then exposed to an atmosphere of N_2_O. After work-up, the heterocycles 39 or 40 were obtained (Scheme 15). The substrate scope for these transformations was found to be broad, and the heterocycles were obtained in synthetically useful yields. The authors propose that the reactions are initiated by the reaction of N_2_O with an aryl lithium species, leading to the formation of a diazotate. The products are then generated by N–N bond formation and liberation of Li_2_O.

**Scheme 16:**
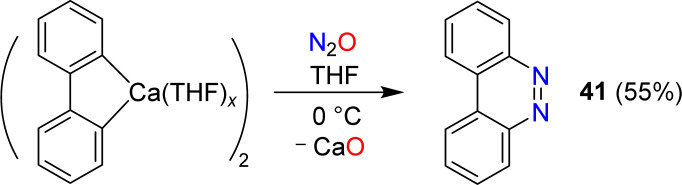
Synthesis of benzo[*c*]cinnoline.

Azobenzene can be obtained by reaction of phenylcalcium iodide and N_2_O (see Section 6). A related diazo transfer reaction was observed when a dimeric biphenylcalcium complex was mixed with N_2_O.^87^ Benzo[*c*]cinnoline (41) was obtained in 55% yield (Scheme 16).

**Scheme 17:**
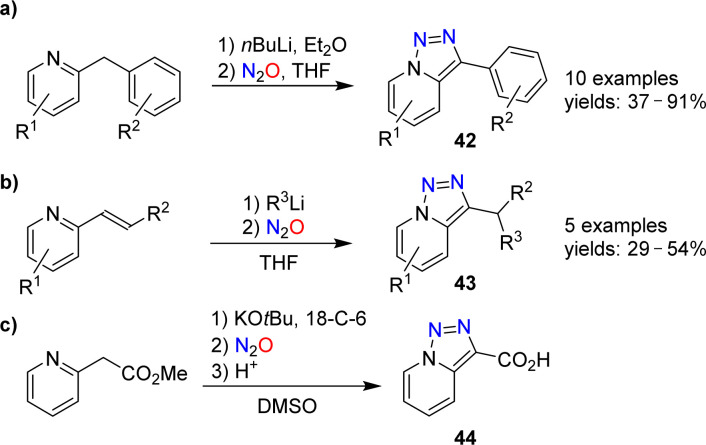
Syntheses of triazolopyridines.

Triazolopyridines are valuable starting materials for heterocycle synthesis.^88^ Our group has shown that triazolopyridines can be prepared using nitrous oxide.^89,90^ A range of lithiated 2-benzylpyridines could be converted into triazolopyridines of type 42 upon reaction with N_2_O (Scheme 17a).

The diazo transfer reaction can be combined with a C–C bond-forming reaction. Heterocycles of type 43 were obtained by coupling of organolithium reagents with 2-vinylpyridines, followed by N_2_O-induced triazole formation (Scheme 17b). The carboxylic acid 44, on the other hand, was prepared in 89% yield by deprotonation of methyl 2-(pyridin-2-yl)acetate, followed by reaction with N_2_O and hydrolysis (Scheme 17c).

## 6. Synthesis of azo compounds

**Scheme 18:**
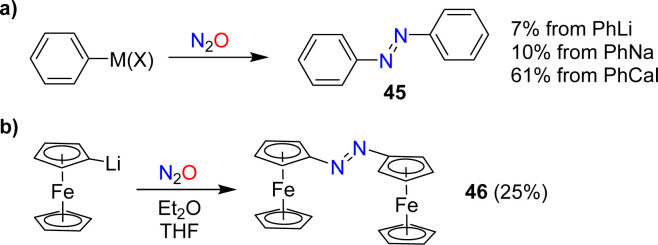
Synthesis of azobenzene and azo-bridged ferrocene.

In 1953, Beringer, Farr and Sands published a study describing reactions of organolithium reagents with N_2_O.^91^ For phenyllithium, they observed a mixture of products, including biphenyl, triphenylhydrazine, phenol, and a small amount (7%) of azobenzene 45 (Scheme 18a). Similar products were found by Meier when using PhNa instead of PhLi. Meier also showed that PhCaI can be converted into azobenzene.^92^ In this context, it is worth noting that aryl Grignard reagents are largely inert towards N_2_O.^92,93^

In 1995, the reaction between PhCaI and N_2_O was re-investigated by Hays and Hanusa.^94^ By optimizing the procedure, they were able to obtain azobenzene with a yield of up to 61% (Scheme 18a). However, they noted difficulties in obtaining reproducible results.

Azo-bridged ferrocene (46) was obtained in 25% yield by reaction of lithiated ferrocene with N_2_O (Scheme 18b).^95^ A related reaction was used to synthesize azo-bridged ferrocene oligomers.^96^

**Scheme 19:**
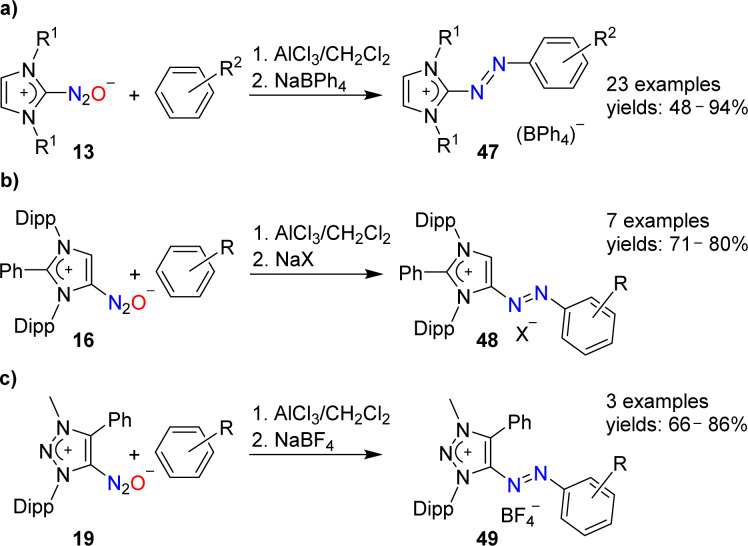
Synthesis of cationic azo dyes from NHC–N_2_O adducts.

N-Heterocyclic carbenes are able to form stable covalent adducts with N_2_O (see Section 2 and Scheme 7). In the presence of AlCl_3_, adducts of type 13 can be coupled to arenes (Scheme 19a).^97^ The resulting azo compounds are of interest as dyes. They are produced industrially *via* different routes, and they have found diverse applications.^98^ The N_2_O-based methodology has a good scope with regard to the arene coupling partner, and NHC(N_2_O) adducts with alkyl or aryl wingtip groups can be employed in these reactions.

Azoimidazolium dyes with *N*-aryl substituents were found to display interesting chemistry. Upon reduction, stable aminyl radicals were formed.^99^ Moreover, they can be used as precursors for mesoionic carbene ligands.^100^

The AlCl_3_-mediated coupling chemistry can be extended to N_2_O adducts of mesoionic carbenes.^101^ Azoimidazolium salts of type 48 were formed by coupling of arenes with 16 (Scheme 19b), whereas azotriazolium salts (49) were obtained from 19 (Scheme 19c).

**Scheme 20:**
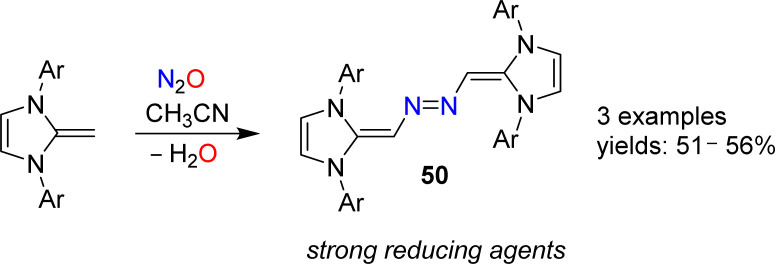
Synthesis of azo-bridged N-heterocyclic olefins.

N-Heterocyclic olefins (NHOs) display a highly polarized exocyclic C=C double bond, making them strong bases and nucleophiles.^102^ In 2019, our group reported that NHOs with Dipp, mesityl or xylyl wingtip groups are able to activate N_2_O.^103^ When a solution of the respective NHO in acetonitrile was subjected to an atmosphere of N_2_O, azo-bridged dimers of type 50 were obtained (Scheme 20). The yields were not high (∼50%), but the products were easily isolated because they crystallized from solution. Reactions between NHOs and N_2_O can also give diazoolefins, and more details about these transformations are given in the next section.

The dimers 50 were found to be very strong electron donors, with first oxidation potentials between −1.32 and −1.38 V (*vs.* Fc/Fc^+^). Upon reduction, stable radical cations or dicationic imidazolium salts were obtained.^103^

## 7. Synthesis of diazo compounds

**Scheme 21:**
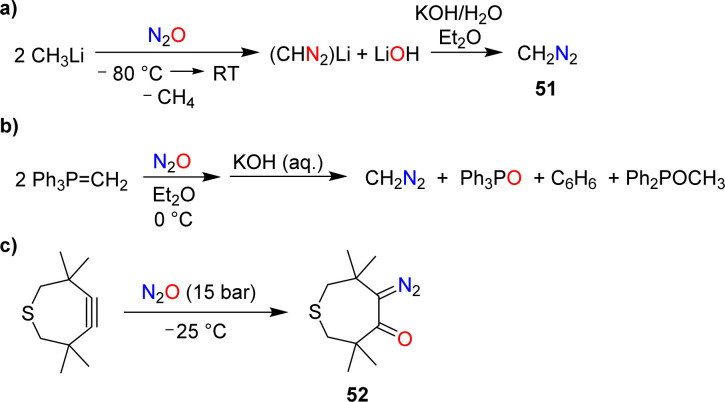
Synthesis of diazomethane (a and b) and addition of N_2_O to a cyclic alkyne (c).

The reaction of N_2_O with methyllithium was first investigated by Müller and coworkers.^104^ They found that diazomethane (51) was formed after basic workup (Scheme 21a). A yield of 70% was obtained under optimized conditions.^105^

The formation of diazomethane was also evidenced in reactions of the ylide Ph_3_P=CH_2_ with N_2_O (Scheme 21b).^106^ However, the yield of CH_2_N_2_ in this transformation was low (20–25%).

During their investigations about reactions of cyclic alkynes with N_2_O, Banert and Plefka were able to isolate the diazo compound 52 in 95% yield (Scheme 21c).^85^ Upon warming to room temperature, loss of dinitrogen was observed, resulting in a mixture of compounds.

**Scheme 22:**
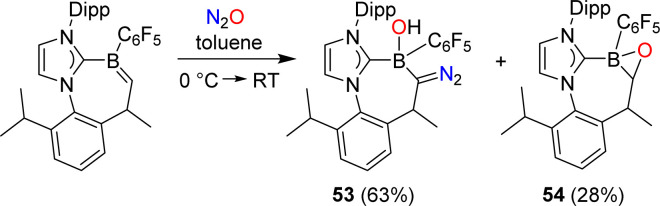
Synthesis of diazo compound 53.

Erker and coworkers have investigated the reactivity of a carbene-stabilized boraalkene.^107^ The reaction with N_2_O gave a mixture of the diazo compound 53 and the oxaborirane 54 (Scheme 22). The authors propose that the compounds are derived from the same intermediate, a (2 + 3) cycloaddition product of the starting material and N_2_O.

**Scheme 23:**
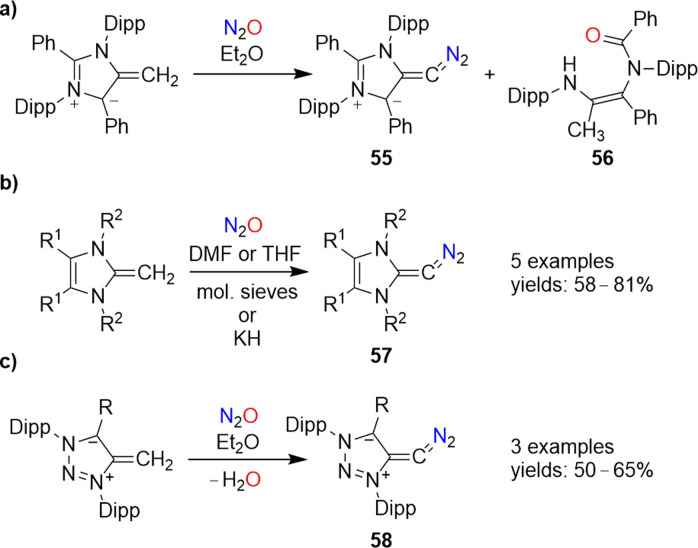
Synthesis of N-heterocyclic diazoolefins.

Diazoolefins of the general formula R^1^R^2^C=CN_2_ (R^1/2^ = alkyl, aryl, or H) are highly reactive compounds, which rapidly lose N_2_.^108^ In 2021, the Hansmann group reported that N_2_O could be used for the synthesis of a room-temperature-stable diazoolefin (diazoalkene).^109^ The reaction of a mesoionic NHO^110^ with N_2_O gave diazoolefin 55 along with amide 56 (Scheme 23a). The diazoolefin could be isolated in 41% yield. A crystallographic analysis of 55 revealed a bent heterocumulene group. The unusual stability of 55 was attributed to both resonance stabilization and polarization of the C–CN_2_ bond.^108*a*,109^

‘Normal’ N-heterocyclic olefins can also react with N_2_O to give diazoolefins of type 57 (Scheme 23b). First examples were published by our group in 2021,^111^ and a new member of this compound class with R^1^ = R^2^ = Me was recently disclosed by Bismuto and coworkers.^112^

The use of triazole-based NHOs allowed access to diazoolefins of type 58 (Scheme 23d).^113^ It is worth noting that both 57 and 58 can also be prepared by using the more conventional diazo transfer reagent *p*-tosyl azide instead of N_2_O.^114^ While nitrous oxide is more atom-economical, the use of *p*-TsN_3_ avoids the formation of the potentially problematic side product water.

**Scheme 24:**
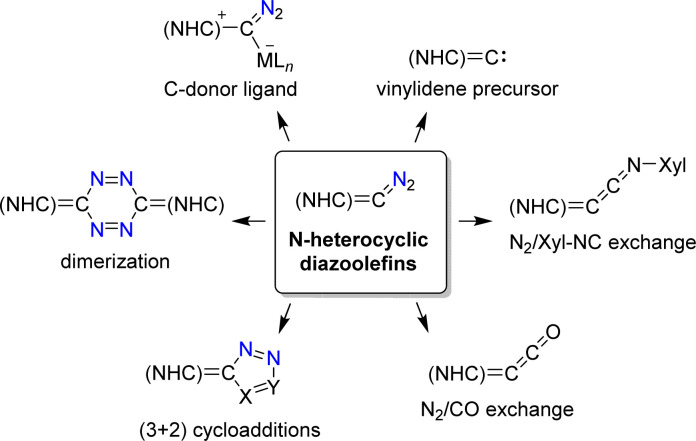
The multifaceted chemistry of N-heterocyclic diazoolefins.

N-Heterocyclic diazoolefins display intriguing chemistry, as evidenced by recent studies (Scheme 24).^108*a*^ They can be used as C-donor ligands for metal complexes^110,111,115,116^ and as precursors for N-heterocyclic vinylidenes (Scheme 24).^112,117–119^ The N_2_ group of N-heterocyclic diazoolefins can be exchanged for isocyanides or for CO to give novel heterocumulenes.^110,113,120^ Cycloaddition reactions with dipolarophiles give pyrazole derivatives,^111,121^ and methanol was found to promote the dimerization of N-heterocyclic diazoolefins.^122^

**Scheme 25:**
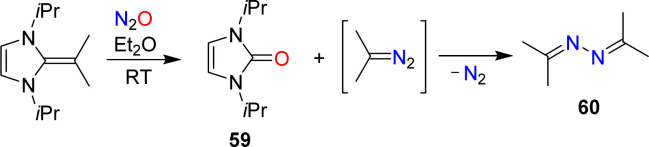
The reaction of a dimethylated N-heterocyclic olefin with nitrous oxide.

The conversion of NHOs to diazoolefins requires the presence of a terminal CH_2_ group. Gellrich and coworkers reported that a *gem*-dimethylated NHO was still able to activate N_2_O.^123^ They observed cleavage of the exocyclic double bond to give the urea 59 along with azine 60 (Scheme 25). The latter was formed by denitrogenative coupling of 2-diazopropane.

**Scheme 26:**
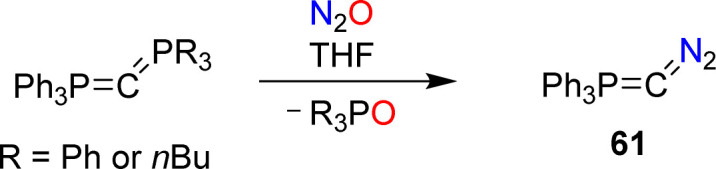
Synthesis of the diazophosphorus ylide 61.

Recently, the Hansmann group reported the synthesis of the diazophosphorus ylide 61.^124^ The diazo compound was obtained by combining carbodiphosphoranes Ph_3_P=C=PR_3_ (R = Ph or *n*Bu) with nitrous oxide (Scheme 26). The ylide serves as a selective transfer reagent for the fragments Ph_3_PC and CN_2_. Furthermore, carbon-atom transfer was observed in reactions of 61 with aldehydes and ketones.

## 8. Conclusions

In synthetic chemistry, nitrous oxide is well known for its ability to act as an oxygen-atom transfer reagent. The present review highlights a distinct reactivity of N_2_O: diazo transfer. Although the application of N_2_O for diazo transfer dates back to the 19th century, it is only in recent years that these reactions have received increased interest.

High-yielding diazo transfer reactions with N_2_O were realized with a range of compounds including lithium amides, metalated arenes and alkanes, N-heterocyclic carbenes, N-heterocyclic olefins, and carbodiphosphoranes. The reactions with these nucleophiles are likely initiated by an attack at the terminal nitrogen atom of N_2_O. In the case of carbenes and amides, the corresponding diazoates could be isolated and characterized. For other nucleophiles, spontaneous N–O bond rupture gave directly nitrogen-containing products.

Several of the compounds described in this review can be prepared by using alternative synthetic procedures. In this case, the advantages and disadvantages of the N_2_O-based methodology must be balanced considering specific constraints (yields, costs, time, availability of N_2_O, *etc.*). For some compounds, nitrous oxide remains the sole viable option for synthesis to date. Alkynyl triazenes, for example, can thus far only be accessed with N_2_O. These activated alkynes are very attractive starting materials for synthetic organic chemistry.^66^

Overall, we hope to have shown with this review that nitrous oxide is more than a simple O-atom donor. Efficient diazo transfer was observed in reactions with a range of carbon- and nitrogen-based nucleophiles. We are confident that there is significant room for further developments. Nitrous oxide has the potential to become a routinely used reagent in synthetic organic and inorganic chemistry.

## Data availability

No primary research results, software or code have been included and no new data were generated or analysed as part of this review.

## Author contributions

A. G. and K. S. co-wrote the manuscript.

## Conflicts of interest

There are no conflicts to declare.
